# AI-Based Response Classification After Anti-VEGF Loading in Neovascular Age-Related Macular Degeneration

**DOI:** 10.3390/diagnostics15172253

**Published:** 2025-09-05

**Authors:** Murat Fırat, İlknur Tuncer Fırat, Ziynet Fadıllıoğlu Üstündağ, Emrah Öztürk, Taner Tuncer

**Affiliations:** 1 Faculty of Medicine, Malatya Turgut Özal University, Ophthalmology, 44090 Malatya, Türkiye; murat.firat@ozal.edu.tr; 2 Faculty of Medicine, Inonu University, Ophthalmology, 44280 Malatya, Türkiye; ilknurtuncer89@gmail.com (İ.T.F.); ziynet.fadillioglu@hotmail.com (Z.F.Ü.); emrah.ozturk@inonu.edu.tr (E.Ö.); 3 Department of Computer Engineering, Firat University, 23119 Elazig, Türkiye

**Keywords:** age-related macular degeneration, anti-VEGF therapy, Siamese network, LayerCam

## Abstract

**Background/Objectives**: Wet age-related macular degeneration (AMD) is a progressive retinal disease characterized by macular neovascularization (MNV). Currently, the standard treatment for wet AMD is intravitreal anti-VEGF administration, which aims to control disease activity by suppressing neovascularization. In clinical practice, the decision to continue or discontinue treatment is largely based on the presence of fluid on optical coherence tomography (OCT) and changes in visual acuity. However, discrepancies between anatomic and functional responses can occur during these assessments. **Methods**: This article presents an artificial intelligence (AI)-based classification model developed to objectively assess the response to anti-VEGF treatment in patients with AMD at 3 months. This retrospective study included 120 patients (144 eyes) who received intravitreal bevacizumab treatment. After bevacizumab loading treatment, the presence of subretinal/intraretinal fluid (SRF/IRF) on OCT images and changes in visual acuity (logMAR) were evaluated. Patients were divided into three groups: Class 0, active disease (persistent SRF/IRF); Class 1, good response (no SRF/IRF and ≥0.1 logMAR improvement); and Class 2, limited response (no SRF/IRF but with <0.1 logMAR improvement). Pre-treatment and 3-month post-treatment OCT image pairs were used for training and testing the artificial intelligence model. Based on this grouping, classification was performed with a Siamese neural network (ResNet-18-based) model. **Results**: The model achieved 95.4% accuracy. The macro precision, macro recall, and macro F1 scores for the classes were 0.948, 0.949, and 0.948, respectively. Layer Class Activation Map (LayerCAM) heat maps and Shapley Additive Explanations (SHAP) overlays confirmed that the model focused on pathology-related regions. **Conclusions**: In conclusion, the model classifies post-loading response by predicting both anatomic disease activity and visual prognosis from OCT images.

## 1. Introduction

Age-related macular degeneration (AMD) is the leading cause of permanent vision loss in individuals over 50 years of age in developed countries [1]. The neovascular form of the disease (nAMD) is characterized by macular neovascularization (MNV) and associated with pathological findings such as subretinal/intraretinal fluid accumulation (SRF/IRF) and, in advanced stages, subretinal fibrosis and atrophy [2]. Currently, the first-line treatment for nAMD is intravitreal anti-VEGF injections (bevacizumab, ranibizumab, and aflibercept) [1]. These treatments suppress abnormal vessel formation and fluid leakage by inhibiting vascular endothelial growth factor (VEGF) [3]. In clinical practice, regardless of treatment protocols (pro re nata, treat-and-extend, or monthly), the decision to continue or discontinue treatment is largely based on optical coherence tomography (OCT) findings and visual acuity measurements [4]. However, this process presents two fundamental clinical challenges: (1) determining the optimal treatment burden for individual patients and (2) accurately estimating visual prognosis [5]. Moreover, in clinical practice, interpreting cases where patients show insufficient visual acuity improvement but no evidence of active disease on OCT is not always straightforward [6].

In recent years, various artificial intelligence approaches have been developed to address these challenges. Romo-Bucheli et al. [7] proposed a deep learning model based on DenseNet and recurrent neural networks (RNNs) to predict treatment requirements in nAMD patients and achieved 64% overall accuracy. This end-to-end model targets monthly injection demand but does not offer clinician-facing explanations linked to OCT regions [7]. Chandra et al. [8] used random forest, support vector machine (SVM), and extreme gradient boosting (XGBoost) algorithms to estimate the number of anti-VEGF injections (AUC: 0.82) and emphasized the critical importance of the first 12 weeks of treatment. Bogunovic et al. [9] predicted treatment burden (AUC: 70–80%) with a machine-learning-based model and demonstrated the importance of combining visual acuity, demographic data, and baseline OCT images. This machine-learning approach predicts injection burden using segmentation-derived features but provides limited explainability and does not address paired pre- and post-treatment analysis [9]. Jung et al. [10] attempted to predict nAMD recurrence using the DenseNet201 architecture and achieved 52.17% accuracy with a single pre-injection OCT image and 53.3% accuracy with four OCT images obtained during three loading injections. This recurrence model uses single-timepoint OCT inputs with modest accuracy but does not relate OCT findings to changes in visual acuity [10]. Moon et al. [11] proposed a GAN-based artificial intelligence model that can accurately select anti-VEGF drugs for patients with neovascular age-related macular degeneration. This GAN-based work focuses on selecting the anti-VEGF agent based on post-treatment anatomy, without modeling visual prognosis or paired pre- and post-scans [11]. Gallardo et al. [12] presented a machine learning algorithm that predicts the need for treatment in cases of nAMD, DME, and retinal vein occlusion (RVO). Gutfleisch et al. [13] developed convolutional neural networks that can distinguish the tissues requiring treatment in nAMD based on SD-OCT. Other studies have developed deep learning models to predict the anti-VEGF response in DME [14,15,16,17]. However, none of these studies have proposed a comprehensive classification model that evaluates both anatomical (OCT) and functional (visual acuity) parameters to support treatment decisions. In this study, a Siamese-network-based model was developed to evaluate the anti-VEGF treatment response in nAMD patients and determine the subsequent treatment course. Our model can simultaneously assess both anatomical activity (presence of SRF/IRF) and functional prognosis (change in visual acuity) by analyzing pre- and post-treatment OCT image pairs. To our knowledge, this approach is the first to be presented in the literature. Our model makes a significant contribution, especially in the management of ambiguous cases that complicate clinical decision-making.

Recent reviews show a shift from single-timepoint classification toward longitudinal and multimodal AI analyses in AMD, with growing attention to robust workflows and external validation [18,19,20,21]. Crincoli et al. summarize AI applications across screening, diagnosis, prognosis, and decision support [19]. Frank-Publig et al. discuss progression assessment and the need for structural and functional biomarkers that enable individualized treatment [20]. Chen et al. outline accountable AI workflows with external validation using AMD as an exemplar [21]. In parallel, biomarker syntheses indicate that OCT features beyond drusen volume carry incremental prognostic value. These include intraretinal hyperreflective foci, shallow irregular RPE elevation, subretinal hyperreflective material, non-exudative subretinal fluid, and nascent atrophy [22,23,24]. Trinh et al. meta-analytically rank high-yield OCT markers for late AMD [22], Gnanaraj et al. validated OCT biomarkers in a 10-year cohort [23], and Nanji et al. quantify baseline OCT features linked to visual outcomes [24]. Together, these updates motivate approaches that integrate anatomical dynamics with functional endpoints rather than relying on static images alone [19,20,21,22,23,24].

Recent prediction studies in nAMD include discrete outcome classifiers and generative models [19,25,26]. Han et al. trained a deep model on serial OCT to predict post-treatment status as dry or non-dry [18]. Chandra et al. combined early clinical and imaging features to forecast two-year visual acuity [25]. Lee et al. used a conditional generative model to simulate twelve-month OCT from baseline and treatment variables and showed that adding post-treatment OCT improves long-horizon predictions [26]. In contrast, our work analyzes paired pre- and post-treatment OCT with a Siamese ResNet-18 and assigns patients to three clinically actionable groups, coupling anatomic change with functional recovery. Accordingly, there remains a gap for methods that analyze paired pre- and post-treatment OCT to capture both anatomical activity and functional prognosis while also providing transparent, clinician-readable explanations, including SHAP-based visualizations, making the method transparent and practical for routine use.

In this study, a Siamese-network–based model was developed to exclusively use OCT and compare pre- and post-treatment scans, providing clear, clinician-readable explanations and making the method transparent and practical for routine use. As a key result, the model achieved 95.4% accuracy and a per-class AUC > 0.95 in our cohort, which compares favorably with prior reports and supports its potential use as an aid in nAMD management.

### Motivation

Deep learning can extract nAMD biomarkers from OCT images and predict patient response to anti-VEGF therapy at a level beyond human judgment. Therefore, a deep-learning-based method was proposed for predicting success and prognosis in anti-VEGF therapy. This study aims to develop a method that can assist in assessing treatment response and demonstrate the effectiveness of the deep learning algorithm during the treatment process. This study is important for the following reasons:

- Monitoring the treatment process of patients requiring intravitreal injections is crucial due to the variability of treatment response.
- Objective criteria are needed to determine anti-VEGF treatment duration in nAMD.
- In clinical practice, discrepancies between OCT findings and visual acuity complicate treatment decisions.

## 2. Data

### 2.1. Dataset

This retrospective study was conducted on 144 eyes of 120 patients treated at İnönü University Turgut Özal Medical Center between January 2021 and March 2025 for nAMD. All patients completed a 3-month bevacizumab loading dose. Prior to the study, approval was obtained from the İnönü University Health Sciences Scientific Research Ethics Committee (Approval No: 2025/8082, approval date 8 July 2025). All OCT images were acquired using the macular radial scanning protocol with the DRI OCT Triton (Topcon, Japan) device, and horizontal section images were used for analysis. The classification and evaluation of the OCT images were performed by three specialist physicians (M.F., İ.T.F., and E.Ö.). Visual acuity was converted from Snellen to logMAR.

### 2.2. Patient Classification

Treatment response was determined based on OCT findings and visual acuity change at 3 months as follows [27]:

- Class 0 (*n* = 59): Presence of SRF and/or IRF on OCT (active disease).
- Class 1 (*n* = 45): Absence of fluid on OCT + ≥0.1 logMAR improvement (good response).
- Class 2 (*n* = 40): Absence of fluid on OCT + <0.1 logMAR change/deterioration (limited response).

**Figure 1:**
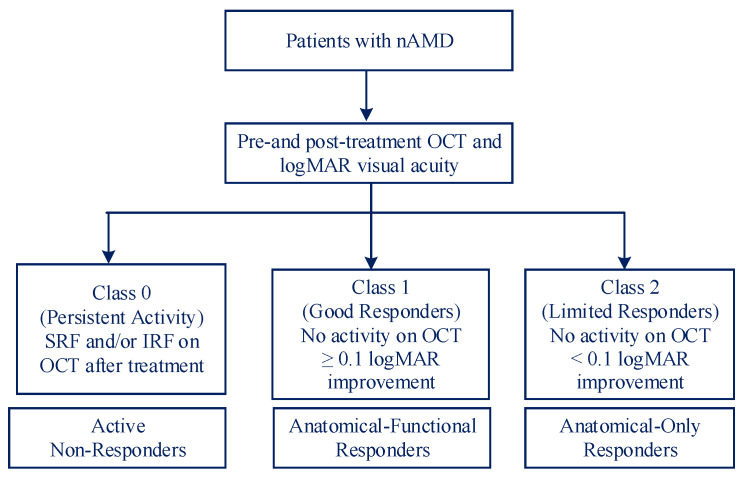
Patient classification.

The complete data collection procedure is given in Figure 1.

Structural OCT data were evaluated using post-treatment images [2]. The evaluation criteria for structural OCT findings are shown in Table 1.

To increase the diversity of the dataset and prevent the AI model from overfitting the data, the images were augmented by applying a 5° rotation to the right and left. A total of 432 OCT images were obtained using the data augmentation process, and Table 2 shows the class distributions in the dataset.

### 2.3. Statistical Analysis

Statistical analyses were performed using SPSS Statistics (22.0, IBM Corp., Armonk, NY, USA). Normality was assessed with the Shapiro–Wilk test. Depending on distribution and study design (independent vs. paired comparisons), appropriate parametric or non-parametric tests were applied with Bonferroni correction where relevant; *p* < 0.05 was considered statistically significant.

Statistical tests were selected based on outcome type (continuous vs. categorical), study design (paired vs. independent), and normality. Continuous data were analyzed with *t*-tests/ANOVA or their nonparametric counterparts (Mann–Whitney U/Kruskal–Wallis; Wilcoxon for paired data). Categorical data were analyzed with χ^2^ or Fisher’s exact test, and multiple comparisons were adjusted using Bonferroni correction where relevant.

## 3. Proposed Method

This section details a Siamese-network-based AI model that classifies post-loading response at 3 months.

**Figure 2:**
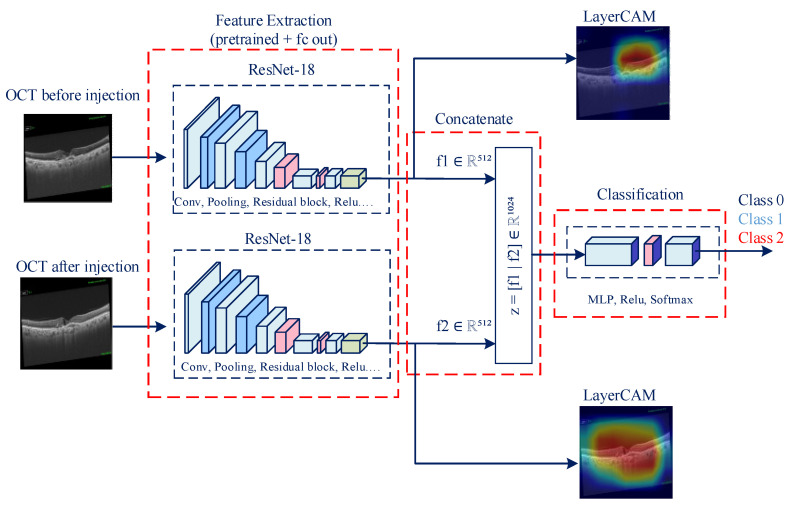
Framework of the proposed artificial-intelligence-based model.

Siamese networks are deep learning architectures developed for comparing text and image data [28,29]. A Siamese network has two identical branches that share weights and process a pair of inputs in parallel. In our setting, the pre- and post-treatment OCT images pass through these branches to produce feature embeddings, which are subsequently combined and fed to a small classifier. This design emphasizes changes between time points rather than absolute appearance and is suitable for evaluating classification. These networks require less labeled data during training due to their robustness to noise and distortions in the data. The Siamese network consists of two identical CNN (backbone) networks, such as ResNet-18, with shared weights. Fixed-size feature vectors can be obtained, extracted, and encoded from the input image pairs. This feature makes it an ideal model for evaluating different image pairs. Figure 2 shows the framework of the proposed model, which consists of feature extraction, feature fusion, and classification stages.

The CNN received only paired OCT B-scans (baseline and 3 months). VA values were used exclusively to construct the response label (Class 1: dry + ΔVA ≥ 0.1 logMAR; Class 2: dry + ΔVA < 0.1 logMAR) and were not fed to the network as inputs. This design keeps the predictions image-driven, links the learned representation to clinically meaningful functional change, and avoids information leakage from VA.

Let *x*_1_ ∈ *R*^3×*H*×*W*^ be the OCT image obtained when the patient presented to the clinic and *x*_2_ ∈ *R*^3×*H*×*W*^ be the OCT image obtained after the anti-VEGF loading therapy (approximately 3 months later). Each image was fed into the ResNet-18 model as input, and feature vectors were obtained from the global average pooling layer. Feature maps are crucial for determining which areas in the image should be considered during the model’s classification process and for ensuring image explainability. The extracted feature vectors, *f*_1_ ∈ *R*^512^ and *f*_2_ ∈ *R*^512^ were combined to obtain a 1 × 1024 *z* vector (Equation (1)). The || operator in the equation enables horizontal concatenation of feature vectors. The resulting *f*_1_ ∈ *R*^512^ and *f*_2_ ∈ *R*^512^ feature vectors (embeddings) are used for both classification and for obtaining LayerCAMs from input images.

(1) $$z=\left[f_{1}||f_{2}\right]\in R^{1024}$$

In the classification process, the feature vector z was given as input to a two-layer multi-layer perceptron (MLP) network. These layers are Linear + ReLU (Equation (2)) and Linear layers (Equation (3)).

(2) $$h=ReLU\left(W_{1}z+b_{1}\right)\in R^{256},\;W_{1}\in R^{256\times 1024},b_{1}\in R^{256}$$

(3) $$l=W_{2}h+b_{2}\in R^{3},W_{2}\in R^{3\times 256},b_{2}\in R^{3}$$

The raw scores *l_i_* (*i* = 0,1,2) obtained from the linear layer were given as input to the SoftMax layer, and class probabilities were calculated (Equation (4)).

(4) $${\hat{y}}_{i}=\frac{exp\left(l_{i}\right)}{\sum_{j=1}^{3}exp\left(l_{i}\right)}$$

Adam optimization was used to minimize the errors occurring in the training process and the error change was determined by the cross-entropy loss function to show the real class labels (Equation (5)).

(5) $$L\left(\hat{y},y\right)=-\sum_{i=1}^{3}y_{i}log\left({\hat{y}}_{i}\right)$$

To limit overfitting, we used patient-level splits with stratified 5-fold cross-validation on the training set, light data augmentation, and a held-out test set.

Hyperparameters were selected with a small grid search using stratified 5-fold cross-validation on the training set, choosing the configuration with the highest mean validation accuracy across folds. Training ran for 40 epochs (fixed a priori). No early stopping, dropout, or weight decay were used. Random parameters were fixed for repeatability.

LayerCAM was used to determine which areas in the image each ResNet-18 model focused on and to ensure explainability of the treatment process. The gradient was calculated using the activations obtained from the final convolution layers of the ResNet-18 models. Activations indicate the importance of the pixel in the classification. $A_{i,j}^{k}$ is determined by the gradient ∇ = ∂yc∂Ak to represent the activation map at positions *i* and *j* in channel *k*. *y*_c_ determines the output of class *c*.

The calculated gradients determine how much influence each pixel has on the model’s output score. For LayerCAM, the contribution (which indicates how much influence a neuron, channel, or pixel has on the model’s output) was calculated using gradients and activation, as in noted in Equation (6).

(6) $$S_{i,j}^{k}=\frac{\partial y^{c}}{\partial A_{i,j}^{k}}A_{i,j}^{k}$$

We also generated post hoc Shapley Additive Explanations (SHAP) maps to identify image regions that most influenced the predicted class in our Siamese model. SHAP was implemented with an image masker and per-branch predictors so that pre- and post-treatment scans could be explained separately. We additionally performed a factor-level SHAP analysis to relate outer-retinal biomarkers (RPE irregularity, EZ integrity, ORA, scar) to the class labels in fluid-free eyes (Classes 1–2), clarifying how the model separates Class 1 vs. Class 2 beyond fluid. Because the presence of fluid in Class 0 prevents reliable evaluation of outer retinal layers, we focused the explanations on fluid-free Classes 1–2 and quantified the contributions of outer-retinal biomarkers (RPE irregularity, EZ integrity, ORA, and scar). At the factor level, we quantified how tabular clinical/OCT variables contributed to two targets: (i) Class 1 vs. Class 2 and (ii) categorical VA improvement (ΔlogMAR ≤ −0.1 vs. otherwise) within Classes 1–2. Predictors included RPE, EZ, ORA, and scar. For VA improvement only, we additionally included pre-treatment VA, age, and gender. All continuous variables were standardized (z-scored), and models used class-weighted logistic regression. SHAP values were obtained with a linear explainer. We report SHAP summary plots (beeswarm) and, for completeness, standardized logistic coefficients (SLC) and odds ratios (OR) from the factor-level models, targeting explainability where it is most informative (fluid-free Classes 1–2) while acknowledging that Class 0 is chiefly driven by SRF/IRF.

The total contribution was calculated as noted in Equation (7), and the heat value for each location (*i*,*j*) was obtained. The ReLU function in Equation (7) was used to ignore negative contributions and determine that only regions supporting the class were significant. Consequently, the heat maps obtained with LayerCAM were visualized by overlaying the original images.

(7) $$CAM_{i,j}^{c}=\sum_{k=1}^{K}ReLU\left(\frac{\partial y^{c}}{\partial A_{i,j}^{k}}A_{i,j}^{k}\right)$$

## 4. Patient Results

A total of 120 patients (144 eyes) were included in the study. The mean age of the patients was 70.7 ± 7.5 years. In total, 32.5% were female (*n* = 39) and 67.5% were male (*n* = 81). Mean pre-treatment visual acuity was 1.21 ± 0.74 logMAR, and post-treatment visual acuity was 1.02 ± 0.55 logMAR.

Pre-treatment visual acuities and changes in visual acuity (ΔVA) differed significantly between the classes (*p* = 0.013 and *p* < 0.001, respectively). Post hoc analysis revealed a significant difference in pre-treatment VA between the limited and good response classes (*p* = 0.0161), while all pairwise comparisons for ΔVA were significant (*p* < 0.001).

When individual groups were evaluated, significant improvement in visual acuity was observed after treatment in the active disease (*p* = 0.0157) and good response (*p* = 0.0021) classes, whereas a significant decrease was seen in the limited response class (*p* < 0.0001). Patient characteristics by class are shown in Table 3.

When all patients were evaluated, only RPE irregularity showed a statistically significant difference between the classes (*p* < 0.001) among structural OCT findings. This finding suggests that RPE irregularity was more prevalent in the limited response class. No significant differences were found regarding the presence of scarring, EZ defect, or outer retinal atrophy. The structural OCT findings in the good response and limited response classes are shown in Table 4.

In the analysis conducted within the deep learning test subclass, significant differences were found only in terms of RPE irregularity and the change in visual acuity (ΔVA) (*p* = 0.009 and *p* = 0.0004, respectively). RPE irregularity was present in all patients in the limited response class, while only one-third of the good response class exhibited this finding. A significant improvement in visual acuity was observed after treatment in the good response class (*p* = 0.0019), with a mean ΔVA of +0.49 ± 0.334 logMAR. In contrast, no significant change was found in the limited response class (ΔVA = −0.02 ± 0.075, *p* = 0.317). No statistically significant differences were detected between the classes in terms of baseline visual acuity, presence of scar tissue, EZ defects, or outer retinal atrophy.

These findings support that the test subclass adequately represented both response profiles and highlight the potential predictive value of RPE irregularity and changes in visual acuity. Structural OCT findings and visual acuity data for the patients used in the testing subclass are summarized in Table 5.

In fluid-free Class 1 vs. 2 (AUC 0.933), SHAP ranked RPE as the top discriminator, with SLC 1.29 and OR 3.63. Within Classes 1–2 for VA improvement (AUC 0.867), RPE contributed negatively (SLC −1.56, OR 0.21), whereas scar (SLC 0.99, OR 2.70) and worse pre-treatment VA (SLC 0.93, OR 2.53) contributed positively.

## 5. Experimental Results and Discussion

A computer environment with an Intel(R) Core (TM) i7-9750H CPU @ 2.60 GHz (2.59 GHz), 8 GB of RAM, and the Python 3.13.5 programming language was used for training and testing the proposed model. The model’s hyperparameters for the training process were selected as shown in Table 6.

To demonstrate the training process and performance of the proposed model, the loss function change, confusion matrix, ROC curve, LayerCAM, and SHAP analyses were examined. These parameters reveal the model’s performance, including overtraining, misclassification distribution, performance differences between classes, and the areas of the image that contribute most to the classification.

**Figure 3:**
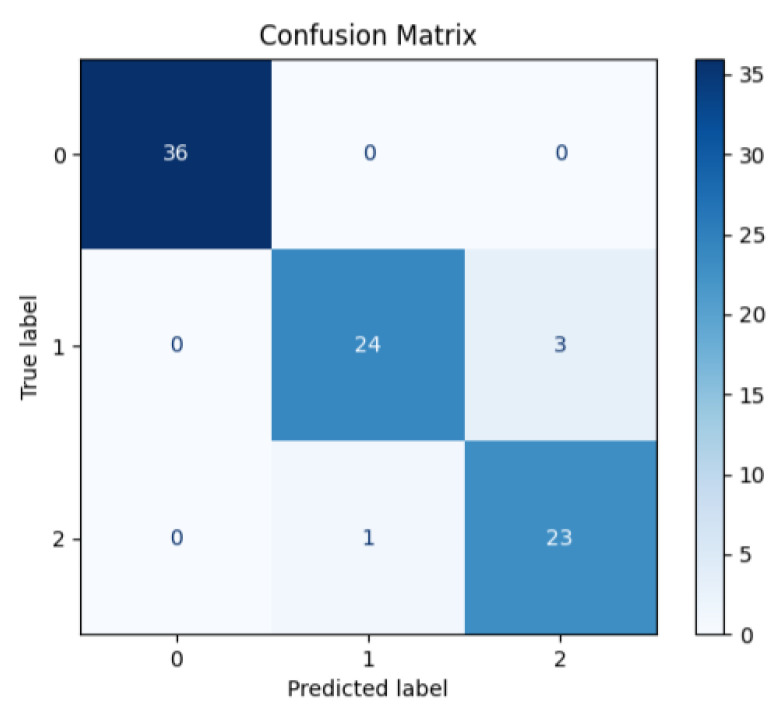
Confusion matrix.

The model’s performance parameters were evaluated using the confusion matrix and the resulting accuracy, F1 score, precision, and recall parameters (Equations (8)–(11)). Additionally, ROC-AUC (receiver operating characteristic–area under the curve) was used to assess the model’s performance on a class-by-class basis. Figure 3 shows the resulting complexity matrix, and Table 7 shows the precision, recall, and F1 score values for each class. The confusion matrix demonstrated successful discrimination between classes. Three images in Class 1 were misclassified as Class 2 and one image in Class 2 was misclassified as Class 1, achieving an accuracy of 95.4%. The average (macro) precision, recall, and F1 score were 0.948, 0.949, and 0.948, respectively. These results demonstrate that the distinction between classes is performed with high accuracy. As an overfitting check, training loss steadily declined and stabilized by epoch 40, indicating no instability; results are reported on a held-out test set.

(8) $$Accuracy=\frac{TP+TN}{TP+FP+TN+FN}$$

(9) $$Recall=\frac{TP}{TP+FN}$$

(10) $$Precision=\frac{TP}{TP+FP}$$

(11) $$F_{1}=\frac{2TP}{2TP+FN+FP}$$

**Figure 4:**
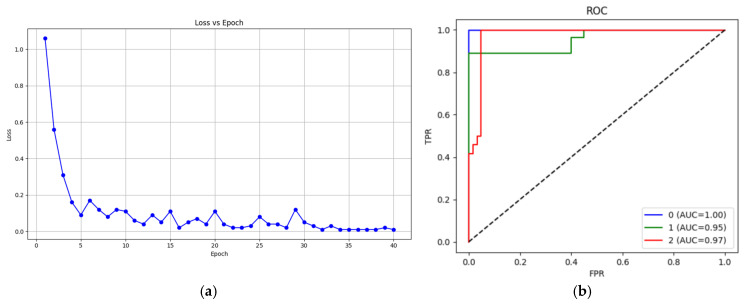
(**a**) Loss function change and (**b**) ROC curve.

The loss function was examined to demonstrate the completion of the training process and learning, and the ROC curve change was examined to observe the distinction between classes. The loss graph change obtained during training process is shown in Figure 4a, and the ROC curve change is shown in Figure 4b. As seen in Figure 4a, the loss value showed a rapid decrease after the first five epochs. Although the loss function fluctuated in subsequent epochs, it stabilized after the 30th epoch. This result indicates that the training was completed successfully.

As shown in Figure 4b, the area under the curve for all three classes is greater than 0.95, suggesting that the model distinguishes each class well. The ROC curves for each class are near the upper left corner. This indicates that the model operates with high sensitivity and a low error rate.

**Figure 5:**
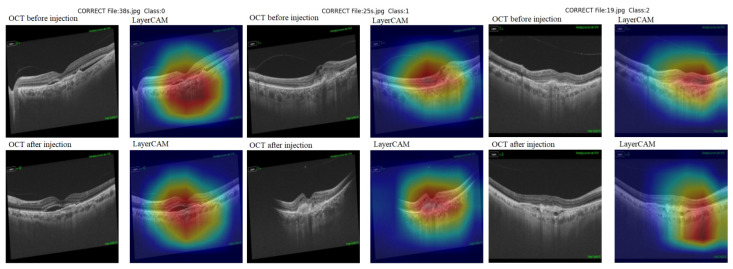
Correctly classified images with LayerCAM heat maps. LayerCAM highlights class-relevant OCT biomarkers (SRF, IRF, fibrovascular PED).

**Figure 6:**
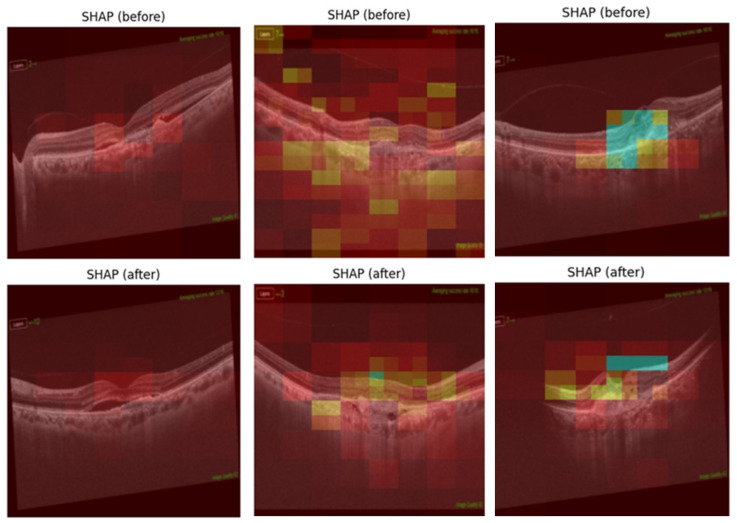
Correctly classified images with SHAP overlays.

**Figure 7:**
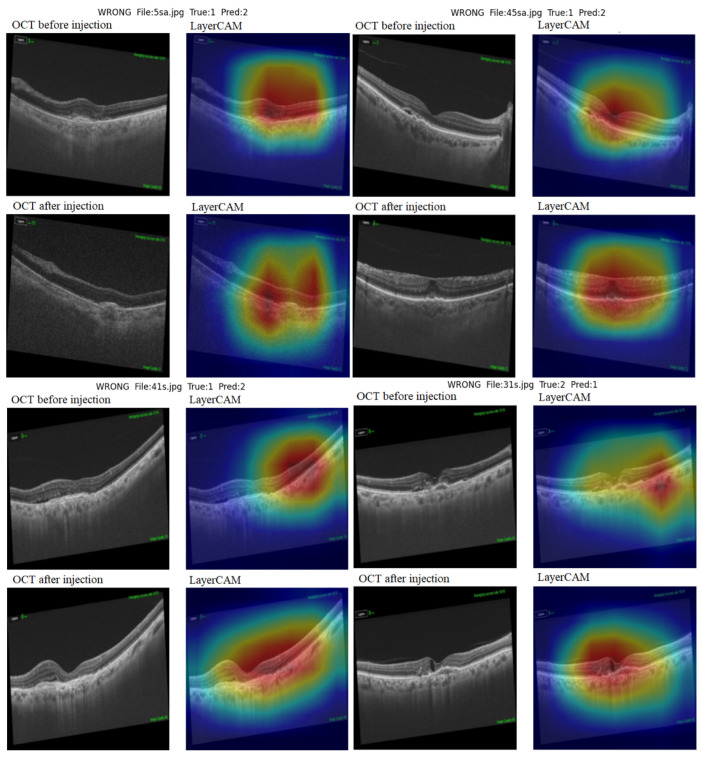
Examples of misclassified images.

**Figure 8:**
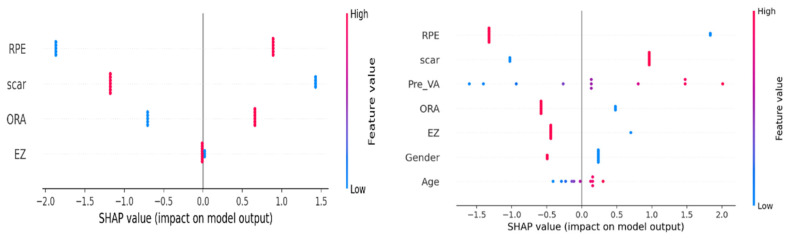
SHAP beeswarms. (**Left**) Class 1 vs. 2: RPE irregularity drives Class 2; scar pushes toward Class 1. (**Right**) VA improvement (ΔlogMAR ≤ −0.1): RPE irregularity lowers improvement; scar and higher pre-treatment VA increase it. ORA and EZ have minimal impacts.

Layer class activation map (LayerCAM) was used to aid clinical evaluation and to demonstrate explainability. LayerCAM generates a heat map using the target gradients of the last convolutional layer in ResNet-18. Warm colors highlight regions that required attention during the decision process. Examples of correct and incorrect detections and their LayerCAM heat maps are shown in Figure 5 and Figure 7. For the same cases in Figure 5, we also provide SHAP overlays that emphasize pathology-related regions on OCT (Figure 6). These views are consistent with LayerCAM, highlighting macular fluid or scar pre-treatment and more focal regions post-treatment. Beyond visualization, we quantified factor-level explainability with SHAP. In fluid-free Class 1 vs. 2, the beeswarm shows RPE irregularity as the strongest discriminator, followed by scar, ORA, and EZ. Within Classes 1–2, the VA improvement beeswarm shows RPE as the most inhibitory factor for improvement, while scar and worse pre-treatment VA push predictions toward improvement, with smaller effects from ORA, EZ, gender, and age (Figure 8).

This artificial intelligence model, developed to support clinical decision-making after anti-VEGF loading in nAMD treatment, was implemented using a ResNet-18-based Siamese architecture. Classification was performed by comparing pre- and post-treatment OCT image pairs for each patient. As can be seen in Figure 5, the model successfully focused on areas of interest in patients’ OCT images that should be evaluated for pathology. The hot regions in the correct classifications correspond to significant pathological structures such as SRF areas and subfoveal scars, demonstrating that the model focused on clinically significant regions during the decision-making process.

Three of the four cases in Figure 6 (top row and bottom left image pair) were classified by the model as limited response when they actually belonged to the good response group. The bottom right image pair, however, was actually in the limited response group and was labeled as a good response.

When the misclassified examples are evaluated, it is seen that the amount of SRF in these patients’ pre-treatment OCT images was quite limited. Examining the LayerCAM maps reveals that the model occasionally failed to focus on pathological areas. Furthermore, the epiretinal membrane (ERM) formation that developed after treatment is noteworthy in the top right example. This is a rare feature in the model training set, which may have contributed to the misclassification. Consistently, SHAP overlays in similar failure modes show low-amplitude, spatially dispersed attributions rather than a single dominant focus, suggesting limited separability when fluid is minimal and the outer retina appears largely similar across classes.

All of these misclassifications occurred between the good response and limited response classes. The lack of a significant difference in structural OCT images between these two classes explains the model’s difficulty. Indeed, classification was based exclusively on visual acuity change in these two classes, leading to confusion when classifying patients with visually similar OCT profiles. Factors such as image quality, class imbalance, and limited sample size may also contribute to misclassification. The model’s classifications show a high degree of overlap with data from both the overall patient population and the test subclass. Although visual acuity change (ΔVA) and RPE irregularity showed statistically significant differences between classes, no significant differences were found for scarring, EZ defects, or outer retinal atrophy. The fact that the good response and limited response groups had similar structural OCT parameters demonstrates that the model can identify microstructural differences indistinguishable by the human eye. SHAP factor analysis confirmed that, in fluid-free Classes 1 vs. 2, RPE was the top discriminator. However, for VA improvement within Classes 1–2, pre-treatment VA and RPE irregularity dominated, with smaller effects obtained from other factors. Clinically, Class 0 separates easily due to SRF/IRF. When fluid is absent, separation depends on outer-retinal integrity and baseline vision. With minimal SRF or rare patterns (e.g., ERM), the model may confuse good and limited responders.

Prior work consistently shows that baseline visual acuity is a leading predictor of post-treatment outcomes in nAMD, aligning with our finding that pre-treatment VA dominates the VA improvement model [30]. Beyond this, comprehensive syntheses indicate that the integrity of outer retinal bands (EZ, ELM) correlates with visual function and treatment response (24). Our SHAP analysis further suggests that, when fluid is absent (Classes 1–2), RPE irregularity is an important biomarker for determining whether visual improvement exceeds the 0.1 logMAR threshold. Although RPE abnormalities are recognized in AMD pathobiology and treatment courses [31], their role in fluid-free prognostication warrants confirmation in larger cohorts. Unexpectedly, scar presence predicted greater VA improvement. This may reflect worse baseline logMAR or more intensive therapy, or it may indicate that our scar label partly captures more post-treatment fibrosis with fluid resolution.

Unlike similar nAMD and anti-VEGF-treatment-focused artificial intelligence studies in the literature, our study is one of the limited number of approaches that evaluate both anatomical and functional data together (Table 8). Our three-class, change-focused model compares well with recent binary predictors [18,25] and generative long-term models [32], showing a higher per-class AUC and clearly linking structural changes to visual outcomes. Existing studies typically classify patients after anti-VEGF treatment based exclusively on the presence of fluid or changes in visual acuity [7,13,32,33,34,35]. Using post-treatment OCT, as shown by Lee et al. [26], supports our decision to analyze paired pre- and post-treatment images. Compared with the study by Chandra et al. [25], which forecasts two-year visual acuity from early features, our method provides an immediate post-loading classification label mapped to treatment. Compared with the study by Han et al. [18], our study extends beyond a dry or non-dry endpoint to three clinically actionable classes. Unlike Lee et al. [26], our method yields an interpretable category without simulating future anatomy. However, in our study, many patients with fluid findings after treatment showed greater visual improvement compared to those without, demonstrating that classification based solely on visual acuity change may be insufficient for treatment decisions. These differences align with recent guidance on robust AI workflows [19,20,21] and with biomarker syntheses showing prognostic value beyond drusen [22,23,24]; they also support a combined anatomy–function strategy that better matches post-treatment clinical decision-making.

Although the presence of fluid is generally considered an indicator of active disease and supports the decision to continue treatment, the absence of fluid does not always result in significant visual improvement, complicating the decision-making process. In fact, in our study, similar OCT findings were found in classes without fluid after treatment; however, differing visual responses were observed. This demonstrates that different functional outcomes accompanied by similar structural images are difficult to explain clinically and create uncertainty in the decision-making process. Our model’s greatest contribution in this regard is its ability to provide objective support to clinicians regarding post-treatment visual acuity changes and prognosis expectations in patients with no signs of active disease on OCT findings.

Another method for classifying nAMD patients after anti-VEGF treatment is to monitor treatment markers such as fluid using segmentation-based analysis [36,37]. However, segmentation-based approaches are limited to visible, distinct structures and can overlook microstructural details [35]. Therefore, in our study, a segmentation-free analysis approach was adopted, and our deep learning model was designed to work directly on raw OCT images. This provides a more holistic and integrated assessment capable of recognizing structural differences at the microscopic level. Table 8 presents a comparison of the proposed model with the literature.

The model’s potential for clinical use is particularly evident in nAMD patients undergoing PRN (pro re nata) protocols. Unnecessary injections can be avoided in patients considered to be in the limited response class (Class 2) after loading treatment, while patients in the good response class (Class 1) can be monitored without treatment. Patients in the non-responsive class (Class 0) can continue treatment. This approach can provide a cost-effective, personalized treatment strategy. PRN retreatment rules commonly rely on OCT-detected fluid, a loss of at least five ETDRS letters, or new hemorrhage, as established in the CATT and IVAN protocols [38]. They are also consistent with findings from the FLUID study, which showed that limited subretinal fluid can be tolerated under treat-and-extend regimens without compromising vision, thereby allowing longer treatment intervals [39].

From a service and patient-burden perspective, treat-and-extend (T&E) regimens generally require fewer clinic visits than PRN and yield equal or better visual outcomes in real-world settings [40], whereas economic modeling shows that strategies that maintain effectiveness with fewer visits or injections can be cost-effective or even cost-saving [39]. In this context, our Class 2 (limited response) label helps identify eyes in which observation is reasonable despite dry anatomy and modest functional gains enabling fewer injections without sacrificing outcomes.

The model was developed to support treatment decisions after anti-VEGF loading therapy in patients with wet macular degeneration. The model’s ability to provide decisions by evaluating visual and anatomical data together suggests that it can support personalized treatment planning in the post-treatment period. This aligns with recent advances in AI-driven diagnostics that combine multimodal integration with interpretable modeling to enable personalized care [41,42]. Furthermore, its ability to accurately classify patients with similar structural damage into different classes demonstrates its ability to access details beyond the human eye. In this regard, the model can be a tool that facilitates clinical decision-making and contributes to personalized patient management.

This study has several limitations. The relatively small number of patients and limited data diversity may limit generalizability. The synthetic images used in the data augmentation process may not fully represent clinical reality. Furthermore, because the model was trained for only three months after treatment, it is still insufficient for long-term prognosis prediction. Finally, using OCT images from a single device limits the ability to test the model’s cross-device validity. Images from other devices may differ, so performance may not transfer directly. We view the results as device specific and will validate them using multiple devices/sites with harmonization before clinical use. Our sample size is modest and from a single center, so external validation across devices and populations is needed to confirm generalization.

In future work, we plan to expand the dataset through multi-center collaboration and include scans from different OCT devices, followed by external validation. We will extend the current paired scan design to longitudinal sequences to better capture disease dynamics and explore domain adaptation and self-supervised pretraining to improve generalization with limited labels. We also plan to evaluate model calibration and uncertainty and conduct a prospective study to assess clinical impact.

## 6. Conclusions

In this study, we developed a Siamese network model that uses only OCT to assess anti-VEGF treatment response in nAMD by combining anatomical activity (SRF/IRF) and functional prognosis (change in visual acuity) from pre- and post-treatment scans. The model achieves high accuracy and provides clear, clinician-readable explanations that contextualize the predictions, especially in ambiguous cases. Although our dataset is modest and from a single center, we reduced overfitting with patient-level splits and cross-validation. In future work, we will expand to multi-center and multi-device cohorts, perform external validation, extend to longitudinal sequences, and evaluate calibration and uncertainty to confirm generalizability and clinical impact. SHAP analyses highlight that fluid-free cases require outer-retinal biomarkers for explainability. Our results emphasize RPE for fluid-free class separation and baseline VA for predicting improvement, providing transparent guidance for post-treatment decisions.

## Figures and Tables

**Table 1:** Structural OCT parameter description.

Feature	Description
Presence of a scar	Hyperreflective and well-defined appearance of the subretinal fibrotic area
Ellipsoid zone (EZ)	Integrity of the foveal EZ line (no loss or mild loss vs. partial or significant loss)
Outer retinal atrophy	Presence of hypertransmission with loss of the EZ and RPE beneath the fovea
RPE irregularity	Border disruption and reflective structure irregularities at the RPE level

**Table 2:** Class distribution of OCT images.

Class Name	Raw Dataset	Augmented Dataset
Class 0	59	177
Class 1	45	135
Class 2	40	120

**Table 3:** Baseline characteristics of the patients according to treatment response.

Characteristic	Active Disease	Limited Response	Good Response	*p*-Value
Pre-VA (logMAR)	1.15 ± 0.71	0.98 ± 0.55	1.51 ± 0.83	0.013 ^1^^a^
Post-VA (logMAR)	1.01 ± 0.55	1.08 ± 0.54	0.98 ± 0.56	0.685 ^1^
ΔVA	0.14 ± 0.43 ^3^	−0.10 ± 0.19 ^4^	0.53 ± 0.43 ^5^	<0.001 ^1b^
Right Eye (*n*, %)	31 (52.5%)	20 (50.0%)	20 (44.4%)	0.712 ^2^
Total Eyes (*n*, %)	59 (41.0%)	40 (27.7%)	45 (31.3%)	

Pre-VA: visual acuity before treatment; post-VA: visual acuity after treatment; ΔVA: change in visual acuity. ^1^ Kruskal–Wallis test, ^2^ Chi-square test, ^3^ *p* = 0.0157 (Wilcoxon signed-rank test for pre- and post-treatment for active disease), ^4^ *p* < 0.0001 (Wilcoxon signed-rank test for limited response), ^5^ *p* = 0.0021 (Wilcoxon signed-rank test for good response). ^a^ Pre-VA post hoc (Bonferroni): limited vs. good response, *p* = 0.0161. ^b^ ΔVA post hoc (Bonferroni): all pairwise comparisons, *p* < 0.001.

**Table 4:** Comparison of structural OCT features between classes.

Feature	Good Response	Limited Response	*p*-Value
Scar	27 (60.0%)	19 (47.5%)	0.1836 ^1^
EZ defect	34 (75.6%)	32 (80.0%)	0.8180 ^1^
Outer retinal atrophy	21 (46.7%)	23 (57.5%)	0.4353 ^1^
RPE irregularity	22 (48.9%)	36 (92.3%)	<0.001 ^1^

^1^ Chi-square test; EZ: ellipsoid zone; RPE: retinal pigment epithelium.

**Table 5:** Comparison of structural OCT and visual acuity characteristics in the test subclass.

Feature	Good Response (*n* = 9)	Limited Response (*n* = 8)	*p*-Value
Pre-VA (logMAR)	1.66 ± 1.00	1.15 ± 0.28	0.186 ^1^
Post-VA (logMAR)	1.17 ± 0.77	1.17 ± 0.31	0.978 ^1^
ΔVA	0.49 ± 0.33 ^4^	−0.02 ± 0.07 ^5^	0.0004 ^2^
Scar	6 (66.7%)	4 (50.0%)	0.637 ^3^
EZ defect	6 (66.7%)	5 (62.5%)	1.0000 ^3^
Outer retinal atrophy	4 (44.4%)	4 (50.0%)	1.0000 ^3^
RPE irregularity	3 (33.3%)	8 (100.0%)	0.009 ^3^

Pre-VA: visual acuity before treatment; post-VA: visual acuity after treatment; ΔVA: change in visual acuity; EZ: ellipsoid zone; RPE: retinal pigment epithelium. ^1^ Unpaired *t*-test, ^2^ Mann–Whitney U test, ^3^ Fisher’s exact test, ^4^ *p* = 0.0019 (paired *t*-test in good response), ^5^ *p* = 0.317 (Wilcoxon signed-rank test in limited response).

**Table 6:** Training parameters.

Parameter	Description
Epoch	40
Batch Size	8
Learning Rate	1 × 10^−4^
Optimizer	Adam
Loss Function	CrossEntropyLoss
Backbone	ResNet-18
Embedding Vector Size	512
Classification Head	Linear(1024 → 256) → ReLU → Linear(256 → 3)
Image Size	224 × 224 px
LayerCAM Destination Layer	backbone.layer4(1).conv2

**Table 7:** Performance parameters obtained as a result of classification.

Class	Precision	Recall	F1 Score
Class 0: Active Disease	1.0	1.0	1.0
Class 1: Good Response	0.960	0.889	0.923
Class 2: Limited Response	0.885	0.958	0.919
Macro Average	0.948	0.949	0.948

**Table 8:** Studies evaluating nAMD anti-VEGF therapy using deep learning without segmentation.

Ref.	Model	Data	Input	Output/ Classes	Performance	Attention Map
Zhao et al., 2021 [32]	SSG-Net	206 paired OCT images (181 patients), 4944 B-scans	Pre-OCT and post-OCT images	Responders (VA increase ≥ 1 line) vs. non-responders (VA decrease or stable)	Accuracy: 84.6%, AUC: 0.83, Recall: 0.692, Specificity: 1.0	Yes
Yeh et al., 2022 [33]	HDF-Net	698 eyes, pre-OCT + demographic data	Pre-OCT + age, gender, BCVA	VA increase ≥ 2 lines vs. <2 lines after 12 months	Accuracy: 93.6%, AUC: 0.989, Sensitivity: 0.933, Specificity: 0.938	Yes
Song et al., 2025 [34]	ResNet50	150 patients, ME secondary to RVO, DR, and CNV, SD-OCT + clinical data	Pre-OCT + age, gender, BCVA, CRT, treatment type	≥5 letters gain vs. <5 letters gain at month 3	Accuracy: 0.9915, AUC: 0.9996	Yes
Romo-Bucheli et al., 2020 [7]	DenseNet	350 eyes (281 patients), 3D SD-OCT volume	Normalized 3D OCT volume	Monthly injections for 6 months = high demand	Accuracy: 63.76%	No
Han et al., 2024 [18]	Deep Learning Fusion Method	2068 SD-OCT images (517 patients, single eye); before injection, after 1st and 2nd	SD-OCT images (before and during treatment)	Dry macula status after 3 injections (binary classification)	Concatenation best; Accuracy: 0.8201, Sensitivity: 0.9148, Specificity: 0.4260, Precision: 0.720	No
Gutfleisch et al., 2022 [13]	3D-CNN	3237 OCT	3D OCT volume (49 B-scans)	(1) Initial treatment decision. (2) Retreatment decision	Exp. 1: AUC: 0.927 Exp. 2: AUC: 0.865	Yes
Feng et al., 2020 [35]	ResNet-50	228 eyes (171 effective, 57 ineffective), pre-OCT	Pre-treatment OCT image	Effective vs. ineffective based on fluid reduction on day 21	AUC: 0.81, Accuracy: 0.72, Recall: 0.78, Specificity: 0.71	No
**Proposed method**	**Siamese Network**	**120 patients (144 eyes)**	**Pre- and post-treatment OCT images**	**Non-responder, good responder, limited responder**	**Accuracy: 0.954, AUC > 0.95 (per class), Recall: 0.949, Precision: 0.948, F1 score: 0.948**	**Yes**

AUC: Area under the curve, BCVA: best-corrected visual acuity, CNN: convolutional neural network, CRT: central retinal thickness, DR: diabetic retinopathy, ME: macular edema, OCT: optical coherence tomography, RPE: retinal pigment epithelium, RVO: retinal vein occlusion, SD-OCT: spectral-domain optical coherence tomography, SSG-Net: self-supervised guided network, VA: visual acuity, 3D-CNN: three-dimensional convolutional neural network, HDF-Net: hierarchical deep fusion network, ResNet: residual network, DenseNet: densely connected convolutional network.

## Data Availability

The data presented in this study are available on request from the corresponding author due to data privacy.
